# Genetic Association between *NFKBIA* and *NFKB1* Gene Polymorphisms and the Susceptibility to Head and Neck Cancer: A Meta-Analysis

**DOI:** 10.1155/2019/6523837

**Published:** 2019-09-12

**Authors:** Lin Li, Zhong-Ti Zhang

**Affiliations:** VIP Department, School of Stomatology, China Medical University, Shenyang 110002, China

## Abstract

**Background:**

The role of the *NFKB1* gene rs28362491 polymorphism and *NFKBIA* gene rs2233406 polymorphism in the development of head and neck cancer (HNC) remains controversial. This meta-analysis was performed to assess the relationship between the gene polymorphisms and HNC quantitatively.

**Methods:**

PubMed, Embase, Web of Science, WanFang Data, and China National Knowledge databases were used to search for eligible articles. The relationship was evaluated by STATA 11.0.

**Results:**

Eight eligible articles were included in our study. Nine case-control studies from the eight included articles were correlated with rs28362491 polymorphism. Four articles were related to rs2233406 polymorphism. Overall, a significant correlation was observed between the rs28362491 polymorphism and a decreased risk of HNCs (OR = 0.76, 95%CI = 0.60‐0.97 for DD vs. II; OR = 0.80, 95%CI = 0.68‐0.95 for DD vs. DI+II). In subgroup analyses, the rs28362491 polymorphism was associated with the risk of nasopharyngeal carcinoma (NC), but not with oral cancer (OC). In addition, no statistical correlation was found between the polymorphism of rs2233406 and HNCs.

**Conclusion:**

rs28362491 polymorphism was significantly associated with the risk of HNCs, especially with NC. Additionally, our results showed that no association was discovered between rs2233406 polymorphism and HNCs.

## 1. Introduction

Head and neck cancer (HNC) is the sixth most common cancer [1], which arises in the oral cavity, pharynx, nasal cavity, and larynx. Appropriately 650,000 new cases of HNCs and 350,000 deaths occur each year worldwide according to the statistics [2]. As a cancer with a high morbidity rate and low survival rate, it reduces patient quality of life [3–5]. Although the primary etiology of HNC is related to human papillomavirus (HPV), smoking, alcohol, and Epstein-Barr virus (EBV) infection [6–10], only a small number of subjects exposed to these risk factors will suffer from a HNC, which suggests that individual genetic susceptibility might also be strongly correlated with the development of HNCs [3, 11, 12].

Nuclear factor-*κ*B (NF-*κ*B), which was identified initially in 1986, acts as an essential transcription factor associated with cell survival, cell growth, cell replication, and cell apoptosis [13–15]. NF-*κ*B also plays an important role in the development of carcinogenesis and tumor cell's resistance to radiotherapy and chemotherapy [16]. The NF-*κ*B family consists of five members: p65/Rel A, p50/p105, p52/p100, c-Rel, and Rel B. The heterodimer of the p65/Rel A and p50/p105 subunits, encoded by the *NFKB2* and *NFKB1* genes, respectively, is the most common form of NF-*κ*B. Moreover, NF-*κ*B activity would be influenced by *NF-κB inhibitor alpha* (*NFKBIA*) [17–19]. Inappropriate expression of NF-*κ*B has been reported in certain human tumors, such as colorectal cancer, breast cancer, colon cancer, multiple myeloma, melanoma, prostate cancer, and Hodgkin's lymphoma [20–24].

The *NFKB1* and *NFKBIA* genes, located on chromosome 4q24 and 14q13, respectively, are associated with the development of cancers, including oral cancer (OC) [25], gastric cancer [26], colorectal cancer [27], and Hodgkin's lymphoma [28]. In recent years, it has been reported that some polymorphisms of the *NFKB1* gene (rs28362491) and *NFKBIA* gene (rs2233406) might be associated with HNCs [25, 29–35]. However, the results are conflicting. It might be because of small sample sizes, publication bias, and different ethnic backgrounds. Therefore, it is necessary to perform a comprehensive meta-analysis with a large sample size and high statistical power to assess the association between the polymorphisms of the *NFKB1* and *NFKBIA* genes and HNCs. Additionally, trial sequential analysis (TSA) was also conducted to minimize random errors and strengthen the reliability of our conclusions.

## 2. Materials and Methods

### 2.1. Selection of Relevant Studies

The meta-analysis was designed and implemented according to the recommendations of the Preferred Reporting Items for Systematic Reviews and Meta-Analyses statement [36].

PubMed, Embase, Web of Science, WanFang Data, and China National Knowledge databases were searched comprehensively prior to May 1, 2019 with the following terms: “Nuclear factor-*κ*B”, or “Nuclear factor kappa B”, or “NF-*κ*B”, or “*NFKB1*”, or “rs28362491”, or “*NFKBIA*”, or “Nuclear factor kappa B inhibitor”, or “rs2233406” AND “carcinoma”, or “cancer”, or “neoplasm”, or “tumor”, or “carcinogenesis” AND “polymorphism”, or “variants”, or “variant”, or “mutation”, or “mutations”, or “polymorphisms” AND “head and neck”, or “HNC”, or “oral”, or “oral cavity”, or “pharyngeal”, or “laryngeal”, or “nasopharyngeal”, or “oropharyngeal”, or “laryngopharyngeal”, or “hypopharyngeal”. Finally, references cited by all the included studies were scanned to identify relevant articles.

### 2.2. Inclusion and Exclusion Criteria

No limitations were imposed on language or publication date. Articles were enrolled if they met all the following criteria: (1) the paper was case-controlled; (2) the paper assessed the risk of HNC and the rs28362491 polymorphism of the *NFKB1* gene and/or the rs2233406 polymorphism of the *NFKBIA* gene; and (3) information provided by the paper was sufficient to estimate the correlation. Articles which satisfied one of the following criteria were excluded: (1) duplicated papers, reviews, editorials, case reports, commentaries, and nonhuman studies; (2) full-text was not found; and (3) no sufficient data were reported.

### 2.3. Data Extraction and Quality Assessment

Two writers collected the information of each enrolled article according to the inclusion and exclusion criteria independently. First author, year of publication, country, ethnicity, genotyping method, source of control, distributions of genotype, and cancer type were fetched. Quality assessment of the enrolled papers in the analysis was conducted by the Newcastle-Ottawa Scale (NOS) criteria [37].

### 2.4. Trial Sequential Analysis (TSA)

Considering that meta-analysis might result in false positive (type-1 error) or false negative (type-2 error) results due to the sparse data [38], TSA was applied to minimize random errors and increase the reliability of our conclusions. In the present study, TSA was conducted with an overall 5% type-1 error risk and 20% type-2 error risk [39]. According to the required information size (RIS) and risk for type-1 error and type-2 error, TSA monitoring boundaries were constructed. If the *Z*-curve crossed with the TSA monitoring boundary before the RIS was reached or total sample size included in the study reached the RIS, a robust conclusion might have been affirmed and further study would be unnecessary.

### 2.5. False-Positive Report Probability (FPRP) Analysis

We calculated the false-positive report probability (FPRP) [40] to assess the significant results. We set 0.5 as the FPRP threshold and assigned a prior probability of 0.1 to detect an odds ratio (OR) of 1.50 for an association with genotypes under investigation. Only a significant finding with a FPRP value < 0.5 was considered a noteworthy result.

### 2.6. Statistical Method

Statistical analysis was performed using STATA 11.0 (StataCorp LP, College Station, TX, United States). The strength of correlation between the gene polymorphisms and HNCs was evaluated through pooled odds ratios (ORs) along with the corresponding 95% confidence intervals (CIs). The ORs and CIs of each gene polymorphism were calculated for the allelic, homozygote, heterozygote, dominant, and recessive genetic models of each article, respectively. The combined ORs were calculated by *Z*-test, and a value of *P* < 0.05 was regarded as statistically significant.

The between-article heterogeneity was estimated by Cochran's *Q*-test and *I*^2^ statistics. If the *P* value in the *Q* − test > 0.10 and *I*^2^ < 50%, the Mantel-Haenszel fixed-effects model was used to estimate the pooled ORs. Otherwise, the DerSimonian and Laird random-effects model was performed to evaluate the correlation between the polymorphisms of the *NFKB1* gene (rs28362491), *NFKBIA* gene (rs2233406), and HNCs. Hardy-Weinberg equilibrium (HWE) in the controls was calculated by the chi-square test [41]. In order to explore the possible heterogeneity among the articles, subgroup analyses according to the genotyping method and tumor type were performed. Sensitivity analysis was conducted by deleting a single article every time in order to assess the reliability of the overall results. Publication bias was detected by Begg's test and Egger's linear regression.

## 3. Results

### 3.1. Article Characteristics

**Figure 1:**
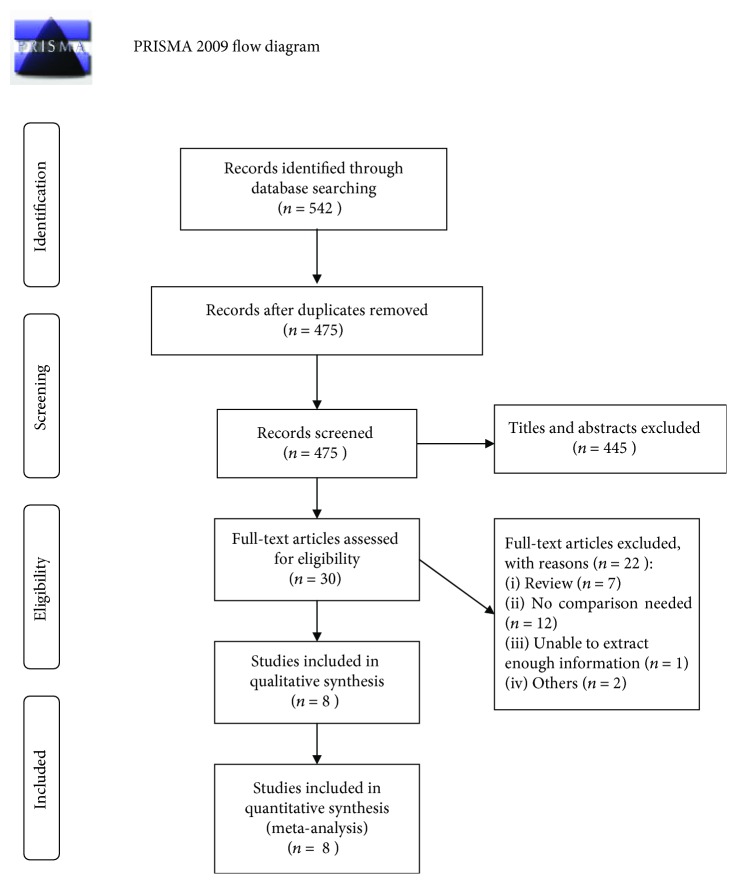
Preferred Reporting Items for Systematic Reviews and Meta-Analyses (PRISMA) flow diagram for a study selection process.

Eight articles [25, 29–35] involving 4434 cases and 4913 controls were finally enrolled in the meta-analysis. The process of study selection is demonstrated in Figure 1.

Nine case-control studies from eight included articles [25, 29–35] were correlated with rs28362491 polymorphism. Among the eight studies, four studies [25, 30, 34, 35] were related to rs2233406 polymorphism. Overall, the meta-analysis included three OC articles, three nasopharyngeal carcinoma (NC) articles, and two HNC articles. All studies showed that the genotype distribution in the controls was conformed to HWE. The detailed information of these enrolled papers is demonstrated in Table 1.

### 3.2. Meta-Analysis Results

The results of the overall analyses and subgroup analyses for gene polymorphisms (rs28362491 and rs2233406) with HNCs are shown in Table 2.

#### 3.2.1. Analysis for rs28362491 Polymorphism

**Figure 2:**
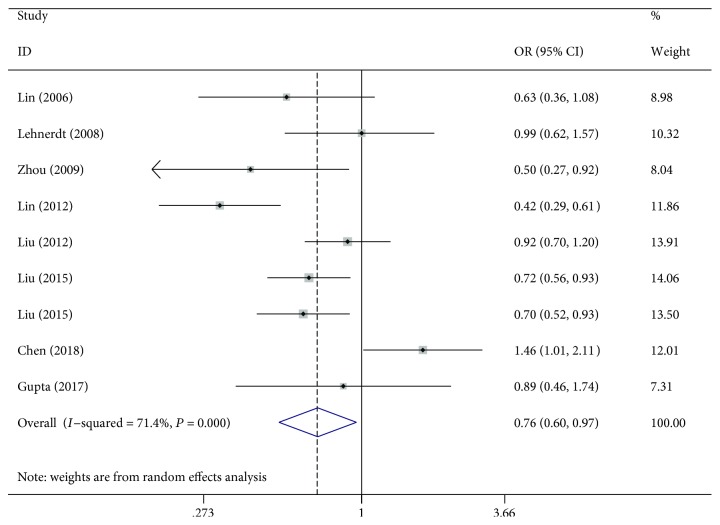
Forest plot for the association of rs28362491 polymorphism and HNC risk under a homozygote genetic model.

For the existence of significant heterogeneity in the *Q*-test (value of *P* in *Q* − test < 0.10 or *I*^2^ > 50%), the random-effects model was performed to analyze the correlation between rs28362491 polymorphism and HNCs under all genetic models. Overall, rs28362491 polymorphism was significantly correlated with a decreased risk of HNCs under homozygote and recessive genetic models (OR = 0.76, 95%CI = 0.60‐0.97 for DD vs. II, Figure 2; OR = 0.80, 95%CI = 0.68‐0.95 for DD vs. DI+II). In subgroup analyses, a significant association was discovered for the polymerase chain reaction-polyacrylamide gel electrophoresis (PCR-PAGE) genotyping method subgroup (OR = 0.74, 95%CI = 0.61‐0.91 for D vs. I; OR = 0.57, 95%CI = 0.38‐0.85 for DD vs. II; OR = 0.67, 95%CI = 0.49‐0.92 for DD+DI vs. II; OR = 0.69, 95%CI = 0.49‐0.98 for DD vs. DI+II), TaqMan genotyping method subgroup (OR = 0.79, 95%CI = 0.69‐0.91 for D vs. I; OR = 0.61, 95%CI = 0.45‐0.83 for DD vs. II; OR = 0.83, 95%CI = 0.69‐1.00 for DI vs. II; OR = 0.75, 95%CI = 0.60‐0.94 for DD+DI vs. II; OR = 0.70, 95%CI = 0.60‐0.83 for DD vs. DI+II), and NC (OR = 0.87, 95%CI = 0.78‐0.96 for D vs. I; OR = 0.75, 95%CI = 0.62‐0.90 for DD vs. II; OR = 0.78, 95%CI = 0.68‐0.89 for DD vs. DI+II).

#### 3.2.2. Analysis for rs2233406 Polymorphism

With respect to rs2233406 polymorphism, the random-effects model was conducted under all genetic models except the recessive model because the statistical heterogeneity between the articles was substantial (value of *P* in *Q* − test < 0.10 or *I*^2^ > 50%). We discovered that there was no relationship between the rs2233406 polymorphism and the risk of HNCs under all genetic models (OR = 1.07, 95%CI = 0.88‐1.30 for T vs. C; OR = 1.17, 95%CI = 0.65‐2.12 for TT vs. CC; OR = 0.99, 95%CI = 0.76‐1.30 for TC vs. CC; OR = 1.01, 95%CI = 0.77‐1.34 for TT+TC vs. CC; OR = 1.11, 95%CI = 0.84‐1.45 for TT vs. CC+TC). In the stratified analyses by genotyping method and tumor type, no association was observed in all genetic models (*P* > 0.05).

### 3.3. Sensitivity Analyses and Publication Bias

In the sensitivity analyses, no substantive change was discovered in the combined ORs after excluding one paper at a time. Egger linear regression tests and Begg's funnel plots were conducted to evaluate publication bias. In all the genetic models, no remarkable publication bias was found by the *P* value in the Egger test (D vs. I: *P* = 0.897; DD vs. II: *P* = 0.788; DI vs. II: *P* = 0.461; DD+DI vs. II: *P* = 0.550; DD vs. DI+II: *P* = 0.996; T vs. C: *P* = 0.071; TT vs. CC: *P* = 0.184; TC vs. CC: *P* = 0.052; TT+TC vs. CC: *P* = 0.066; TT vs. TC+CC: *P* = 0.235) and Begg's funnel plot for rs28362491 and rs2233406 polymorphisms.

### 3.4. TSA and FPRP Analysis Results

**Figure 3:**
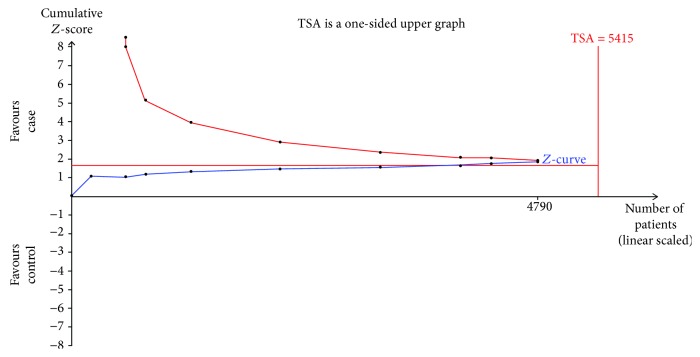
The required information size to demonstrate the association of rs28362491 polymorphism and HNC susceptibility under a homozygote genetic model.

**Figure 4:**
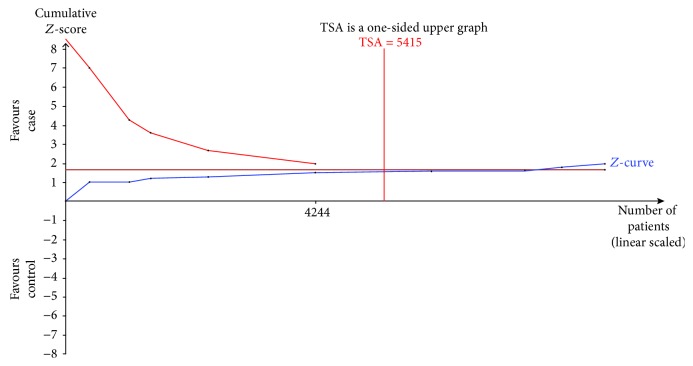
The required information size to demonstrate the association of rs28362491 polymorphism and HNC susceptibility under a recessive genetic model.

Nine trials were used to evaluate the association between the rs28362491 gene polymorphism and the HNC susceptibility. The results of TSA analysis (taking the homozygous model data for example) showed that the cumulative *Z*-curve had not crossed the trial monitoring boundary before the RIS was reached (Figure 3), indicating that the cumulative evidence might be insufficient and further studies are needed to strengthen the conclusion. However, the total sample size of another four genetic models included in the study all reached the RIS, suggesting that the cumulative evidence might be sufficient and further studies would be unnecessary (Figure 4 for the recessive model in rs28362491 gene polymorphism). As for rs2233406, the results of TSA analysis demonstrated that the cumulative *Z*-curve had not crossed the trial monitoring boundary before the RIS was reached under all the genetic models, indicating that further trials are necessary to evaluate the correlation between rs2233406 polymorphism and HNCs (figures were not shown).

The FPRP values for all the discovered significant results are demonstrated in Table 3. For a prior probability of 0.1, the FPRP values were all less than 0.50 in the significant findings, indicating that these significant correlations were noteworthy.

## 4. Discussion

NF-*κ*B plays an essential role in immune response, cell apoptosis, cell proliferation, and the development of cancers [42–44]. Moreover, many inflammatory cytokines, including IL-6, IL-8, and TNF-*α* would be influenced by NF-*κ*B in regulating their biological effects. Therefore, an abnormality in NF-*κ*B function would disturb these various biological behaviors and eventually lead to tumorigenesis [45].

The -94 ins/del ATTG (rs28362491) promoter polymorphism of the *NFKB1* gene encoding p50/p105 subunit of the NF-*κ*B family could modulate the transcription, production, and function of the p50/p105 subunit [45, 46]. NF-*κ*B inhibitor I kappa B (I*κ*B) would inhibit the function of NF-*κ*B by binding to it. *NF-κB inhibitor alph*a (*NFKBIA*), which is the most common protein of the I*κ*B family, is encoded by the *NFKBIA* gene. As a functional polymorphism of the *NFKBIA* gene, the -826 C/T (rs2233406) polymorphism might play an important role in influencing the function of NF-*κ*B. Recently, various articles have explored the relationship between rs28362491 and rs2233406 polymorphisms and HNCs [25, 29–35]. However, the results were inconclusive.

To the best of our knowledge, this is the first meta-analysis to evaluate the association between rs28362491 and rs2233406 polymorphisms and HNCs. In the present study, nine case-control studies from eight eligible papers were enrolled to assess the correlation between -94 ins/del ATTG polymorphism and HNCs. Overall, the results showed that -94 ins/del ATTG (rs28362491) polymorphism was significantly correlated with a decreased risk of HNCs under homozygote and recessive genetic models (OR = 0.76, 95%CI = 0.60‐0.97 for DD vs. II, Figure 2; OR = 0.80, 95%CI = 0.68‐0.95 for DD vs. DI+II). Although the mechanism remains unknown, considering the essential role of NF-*κ*B in the development of cancer, we hypothesize that the possible mechanisms are as follows. NF-*κ*B plays a critical role in various biological pathways, which brings us to the idea that ATTG deletion results in the loss of binding to nuclear proteins, which causes lower promoter activity [47]. The promoter sequence with -94 del allele leads to decreased transcriptional activity and thereby results in decreased p50/p105 expression levels, which plays an essential role in transactivating antiapoptosis genes and restraining cell apoptosis, thereby promoting cellular proliferation. Besides, compared to the -94 ins allele carriers, the p50 in -94 del carriers could form lesser heterodimers with p65 to regulate the inflammatory pathway. Therefore, the -94 del allele might act as a protective role in HNC risk. However, no association between the rs2233406 polymorphism and the risk of HNCs was observed.

In the subgroup analyses of rs28362491 polymorphism based on the genotyping method, a statistically significant correlation was discovered in the PCR-PAGE genotyping method subgroup under allelic, homozygote, dominant, and recessive genetic models and in the TaqMan genotyping method subgroup under all the genetic models, but not in any other genotyping method subgroups. This might be because various genotyping methods would influence the relationship, indicating that it is necessary to confirm a genotyping method with a high specificity and sensitivity to increase the reliability of the results.

Since HNCs include OC, pharyngeal cancer, NC, and laryngeal neoplasm, further stratification analysis by tumor type was performed. When stratified by tumor type, we found that the rs28362491 polymorphism was significantly correlated with a decreased risk of NC under allelic, homozygote, and recessive genetic models. No association was discovered between the gene polymorphism and OC under all genetic models. This might be caused by various microenvironments, because the same gene could play an individual role in different tumor sites [48]. Surprisingly, our results were different from the results presented by four previous meta-analyses [49–52], which suggested that a significant correlation was seen between the rs28362491 polymorphism and OC. These contradictory results might be because all previous analyses only included two studies associated with OC. Therefore, the results suggested by the previous meta-analyses with limited sample sizes were unreliable.

In the subgroup analyses of rs2233406 polymorphism according to the genotyping method and tumor type, there was no relationship between the rs2233406 polymorphism and HNCs.

However, our study has some inevitable limitations. Firstly, some potential articles that have not been published were not enrolled in the present study, so a publication bias might exist. Secondly, our meta-analysis had a relatively small sample size in each subgroup, so the results of the subgroup analyses might not have enough power to identify the association. Thirdly, the environmental factors, such as smoking and alcohol, also play an essential role in the development of HNCs. Unfortunately, subgroup analyses according to smoking or alcohol consumption could not be conducted since there were no sufficient relevant data from most of the enrolled studies. Finally, the results of TSA analysis (taking the homozygous model data for example) showed that the cumulative evidence might be insufficient and further studies would be needed to strengthen the conclusion of rs2233406 polymorphism and the homozygous model in rs28362491 polymorphism. Therefore, further studies with a larger sample size and more information are required to observe the role of rs28362491 and rs2233406 polymorphisms in HNCs.

Despite these shortcomings, our meta-analysis has several highlights. To our knowledge, this present meta-analysis is the first one to conclude a relationship between rs28362491 polymorphism and HNCs and discover that rs28362491 polymorphism is associated with the development of HNCs, especially NC, although there were four meta-analyses [49–52] which focused on the association between the rs28362491 polymorphism and OC. Compared with these, our meta-analysis has the following advantages: Firstly, nine case-control studies from eight eligible papers were enrolled to assess the correlation between -94 ins/del ATTG polymorphism and HNCs; all the previous analyses only included two studies associated with OC. Secondly, the results presented by the four previous meta-analyses [49–52] were different from our results, which suggested that no significant correlation was seen between the rs28362491 polymorphism and OC. These contradictory results might be because all the previous analyses only included two studies associated with OC. Therefore, the results suggested by our meta-analyses with a higher sample size were more reliable than these previous meta-analyses. Thirdly, our study demonstrated that there was no association between the rs2233406 polymorphism and the risk of HNCs, which was not mentioned in previous articles. Finally, in order to make our conclusion more credible, publication bias analysis and sensitivity analysis were performed. Egger's linear regression tests and Begg's funnel plots demonstrated that no obvious publication bias was found. The sensitivity analysis suggested that our conclusions are reliable. Additionally, we performed FPRP analysis, and the results showed that the significant findings of our study are robust. Besides, the meta-analysis clearly shows that the rs28362491 gene polymorphism could be generally applied as a novel prognostic biomarker for HNCs, especially for NC, and it might play a protective role in populations with HNCs, which provides guidance for precise prediction of prognosis and/or therapeutic response.

## 5. Conclusions

In conclusion, this meta-analysis explored that the risk of HNCs was significantly correlated with rs28362491 polymorphism, but not with rs2233406 polymorphism. Moreover, the significant correlation between rs28362491 polymorphism and the susceptibility to NC was identified for the first time. However, more gene-environment and gene-gene interaction papers with larger sample sizes should be addressed to evaluate the relationship between rs28362491 and rs2233406 polymorphisms and HNCs.

## Figures and Tables

**Table 1:** Characteristics of articles enrolled in the meta-analysis.

FA (year)	NOS	Ethnicity	Genotyping method	SC	Cases			Controls			Cancer type	HWE
rs28362491					ins/ins	ins/del	del/del	ins/ins	ins/del	del/del		
Lin (2006)	6	Asian	PCR-PAGE	HB	59	103	50	43	100	58	OC	0.993
Lehnerdt (2008)	7	Caucasian Germany	PCR-RFLP	HB	132	179	53	118	141	48	HNC	0.586
Zhou (2009)	8	Asian	PCR-PAGE	HB	74	67	22	71	90	42	NC	0.177
Lin (2012)	8	Asian	TaqMan	HB	116	246	100	81	271	168	OC	0.099
Liu (2012)	7	Asian	PCR	PB	269	467	170	280	433	193	NC	0.289
Liu (2015)	7	Asian	TaqMan	HB	316	438	152	336	512	224	NC	0.262
Liu (2015)	7	Asian	TaqMan	HB	236	331	117	274	438	195	NC	0.169
Chen (2018)	8	Asian	MassARRAY	HB	124	197	100	163	230	90	OC	0.577
Gupta (2017)	7	Asian	PCR-RFLP	HB	132	162	18	144	146	22	HNC	0.064

rs2233406					CC	CT	TT	CC	CT	TT		
Lin (2012)	8	Asian	TaqMan	HB	351	101	10	438	78	4	OC	0.797
Liu (2012)	7	Asian	PCR	PB	706	185	15	694	201	11	NC	0.402
Liu (2015)	7	Asian	TaqMan	HB	701	188	17	813	244	15	NC	0.492
Chen (2018)	8	Asian	MassARRAY	HB	308	108	9	365	110	8	OC	0.931

FA: first author; SC: source of control; OC: oral cancer; NC: nasopharyngeal carcinoma; HNC: head and neck cancer; HB: hospital-based study; PB: population-based study; HWE: Hardy-Weinberg equilibrium; PCR-RFLP: polymerase chain reaction-restriction fragment length polymorphism; PCR: polymerase chain reaction; PCR-PAGE: polymerase chain reaction-polyacrylamide gel electrophoresis.

**Table 2:** Results of overall and subgroup analyses for gene polymorphisms (rs28362491 and rs2233406).

rs28362491	No	D versus I			DD versus II			DI versus II			DD+DI versus II			DD versus DI+II		
		OR	95% CI	*P* ^(*z*)^	OR	(95% CI)	*P* ^(*z*)^	OR	(95% CI)	*P* ^(*z*)^	OR	(95% CI)	*P* ^(*z*)^	OR	(95% CI)	*P* ^(*z*)^
Overall	9	0.89	0.79-1.00	0.059	0.76	0.60-0.97	0.026	0.95	0.82-1.08	0.419	0.89	0.76-1.05	0.165	0.80	0.68-0.95	0.010
PCR-RFLP	2	1.04	0.89-1.22	0.630	0.96	0.65-1.40	0.813	1.17	0.93-1.48	0.179	1.13	0.91-1.41	0.274	0.88	0.62-1.26	0.495
PCR-PAGE	2	0.74	0.61-0.91	0.004	0.57	0.38-0.85	0.007	0.73	0.53-1.02	0.063	0.67	0.49-0.92	0.012	0.69	0.49-0.98	0.039
TaqMan	3	0.79	0.69-0.91	0.001	0.61	0.45-0.83	0.002	0.83	0.69-1.00	0.047	0.75	0.60-0.94	0.014	0.70	0.60-0.83	<0.001
OC	3	0.86	0.58-1.27	0.457	0.73	0.32-1.65	0.446	0.82	0.56-1.20	0.314	0.79	0.46-1.33	0.369	0.84	0.49-1.46	0.543
HNC	2	1.04	0.88-1.22	0.630	0.96	0.65-1.40	0.813	1.17	0.93-1.48	0.179	1.13	0.91-1.41	0.274	0.88	0.62-1.26	0.495
NC	4	0.87	0.78-0.96	0.007	0.75	0.62-0.90	0.002	0.94	0.81-1.09	0.400	0.87	0.74-1.02	0.096	0.78	0.68-0.89	<0.001

rs2233406	No	T versus C			TT versus CC			TC versus CC			TT+TC versus CC			TT versus CC+TC		
		OR	95% CI	*P* ^(*z*)^	OR	(95% CI)	*P* ^(*z*)^	OR	(95% CI)	*P* ^(*z*)^	OR	(95% CI)	*P* ^(*z*)^	OR	(95% CI)	*P* ^(*z*)^
Overall	4	1.13	0.90-1.42	0.296	1.51	0.99-2.30	0.058	1.08	0.85-1.39	0.523	1.12	0.86-1.44	0.399	1.50	0.98-2.29	0.061
TaqMan	2	1.25	0.72-2.16	0.431	1.68	0.93-3.04	0.084	1.19	0.66-2.12	0.565	1.23	0.68-2.23	0.497	1.67	0.92-3.02	0.089
OC	2	1.39	0.97-1.98	0.074	1.92	0.93-3.98	0.080	1.36	0.99-1.88	0.059	1.40	0.98-2.00	0.062	1.81	0.87-3.75	0.111
NC	2	0.96	0.83-1.10	0.520	1.33	0.79-2.24	0.290	0.90	0.77-1.05	0.180	0.92	0.79-1.07	0.296	1.36	0.81-2.29	0.251

**Table 3:** False-positive report probability values for associations between the rs28362491 polymorphism and the HNC risk.

Variables	OR (95% CI)	*P* ^a^	Power^b^	Prior probability				
				0.25	0.1	0.01	0.001	0.0001
D versus I								
PCR-PAGE	0.74 (0.61-0.91)	0.004	0.839	0.015	0.044	0.338	0.837	0.981
TaqMan	0.79 (0.69-0.91)	0.001	0.991	0.003	0.010	0.098	0.523	0.916
NC	0.87 (0.78-0.96)	0.006	1.000	0.016	0.048	0.355	0.847	0.982

DD versus II								
Overall	0.76 (0.60-0.97)	0.027	0.854	0.088	0.225	0.761	0.970	0.997
PCR-PAGE	0.57 (0.38-0.85)	0.006	0.221	0.073	0.192	0.723	0.963	0.996
TaqMan	0.61 (0.45-0.83)	0.002	0.286	0.017	0.050	0.364	0.853	0.983
NC	0.75 (0.62-0.90)	0.002	0.897	0.007	0.020	0.180	0.688	0.957

DI versus II								
TaqMan	0.83 (0.69-1.00)	0.050	0.989	0.132	0.313	0.833	0.981	0.998

DD+DI versus II								
PCR-PAGE	0.67 (0.49-0.92)	0.013	0.512	0.072	0.190	0.720	0.963	0.996
TaqMan	0.75 (0.60-0.94)	0.013	0.847	0.042	0.117	0.594	0.937	0.993

DD versus DI+II								
Overall	0.80 (0.68-0.95)	0.011	0.981	0.032	0.091	0.524	0.918	0.991
PCR-PAGE	0.69 (0.49-0.98)	0.038	0.576	0.166	0.374	0.868	0.985	0.998
TaqMan	0.70 (0.60-0.83)	0.00004	0.713	<0.001	0.001	0.006	0.054	0.363
NC	0.78 (0.68-0.89)	0.0002	0.990	0.001	0.002	0.022	0.184	0.693

^a^Chi-square test was adopted to calculate the genotype frequency distributions. ^b^Statistical power was calculated using the number of observations in the subgroup and the OR and *P* values in this table.
